# Calycosin as a Novel PI3K Activator Reduces Inflammation and Fibrosis in Heart Failure Through AKT–IKK/STAT3 Axis

**DOI:** 10.3389/fphar.2022.828061

**Published:** 2022-02-21

**Authors:** Xiaoping Wang, Weili Li, Yawen Zhang, Qianbin Sun, Jing Cao, NanNan Tan, Shuangjie Yang, Linghui Lu, Qian Zhang, Peng Wei, Xiao Ma, Wei Wang, Yong Wang

**Affiliations:** ^1^ School of Chinese Medicine, Beijing University of Chinese Medicine, Beijing, China; ^2^ School of Life Sciences, Beijing University of Chinese Medicine, Beijing, China; ^3^ Department of Biochemistry and Molecular Biology, Mayo Clinic, Rochester, MN, United States; ^4^ Department of Cardiovascular Medicine, Mayo Clinic, Rochester, MN, United States; ^5^ Beijing Key Laboratory of TCM Syndrome and Formula, Beijing, China; ^6^ Key Laboratory of Beijing University of Chinese Medicine, Ministry of Education, Beijing, China

**Keywords:** heart failure, calycosin, myocardial fibrosis, inflammation, PI3K–Akt pathway

## Abstract

**Aim:** Inflammation and fibrosis have been shown to be critical factors in heart failure (HF) progression. Calycosin (Cal) is the major active component of *Astragalus mongholicus Bunge* and has been reported to have therapeutic effects on the cardiac dysfunction after myocardial infarction. However, whether Cal could ameliorate myocardial infarction (MI)-induced inflammation and fibrosis and precise mechanisms remain uncertain. The aim of this study is to explore the role of Cal in HF and to clarify the underlying mechanisms.

**Methods:** For *in vivo* experiments, rats underwent left anterior descending artery ligation for heart failure model, and the cardioprotective effects of Cal were measured by echocardiographic assessment and histological examination. RNA-seq approach was applied to explore potential differential genes and pathways. For further mechanistic study, proinflammatory-conditioned media (conditioned media)-induced H9C2 cell injury model and TGFβ-stimulated cardiac fibroblast model were applied to determine the regulatory mechanisms of Cal.

**Results:** In the *in vivo* experiments, echocardiography results showed that Cal significantly improved heart function. GO and reactome enrichment revealed that inflammation and fibrosis pathways are involved in the Cal-treated group. KEGG enrichment indicated that the PI3K–AKT pathway is enriched in the Cal-treated group. Further experiments proved that Cal alleviated cardiomyocyte inflammatory responses evidenced by downregulating the expressions of phosphorylated IκB kinase α/β (*p*-IKKα/β), phosphorylated nuclear factor kapa B (*p*-NFκB), and tumor necrosis factor α (TNFα). Besides, Cal effectively attenuated cardiac fibrosis through the inhibitions of expressions and depositions of collagen I and collagen III. In the *in vitro* experiments, the phosphatidylinositol three kinase (PI3K) inhibitor LY294002 could abrogate the anti-inflammation and antifibrosis therapeutic effects of Cal, demonstrating that the cardioprotective effects of Cal were mediated through upregulations of PI3K and serine/threonine kinase (AKT).

**Conclusion:** Cal inhibited inflammation and fibrosis via activation of the PI3K–AKT pathway in H9C2 cells, fibroblasts, and heart failure in postacute myocardial infarction rats.

## Introduction

Despite recent advances in the therapies of cardiovascular disorders, heart failure (HF) remains a major cause of morbidity and mortality worldwide, which brings great burden on healthcare costs (Rhee and Lavine, 2020). Most often, HF is caused by myocardial infarction (MI) and typically associated with cardiac remodeling (Humeres and Frangogiannis, 2019). Notably, inflammation and fibrosis play crucial roles in the pathophysiology of HF (Bacmeister et al., 2019). Thus, there is an urgent need to develop novel anti-inflammatory and antifibrosis HF therapies.

Inflammation plays an important role in HF. After myocardial infarction, inflammatory response in remote remodeling myocardial segments is activated (Prabhu and Frangogiannis, 2016). Nuclear factor kappa-B (NFκB) is a crucial heterodimeric transcription factor in inflammatory responses, which is regulated by the IκB kinase (IKK) complex (Israël, 2010). Studies have shown that the persistently activated NFκB pathway in HF progression mediated the excessive release of various inflammatory cytokines such as tumor necrosis factor α (TNFα) and interleukin-1 (IL-1) (Wang et al., 2020a). Herein, the IKK-NF-κB pathway is believed to be one of the most attractive targets for HF.

Myocardial pathological remodeling is the major reason for decreased cardiac function HF (Frangogiannis, 2014). Massive sudden loss of cardiomyocytes induced by inflammatory response overwhelms the limited regenerative capacity of the myocardium, resulting in the formation of a collagen-based scar (Prabhu and Frangogiannis, 2016). Along this line, the imbalance of myocardial extracellular matrix (ECM) deposition and degradation promotes the collagen fiber replacement in necrotic myocardial tissue, which contributes to scar tissue accumulation and cardiac fibrosis. Matrix metalloproteinase 9 (MMP-9) could degrade the ECM and plays an important role in the compensation of myocardial fibrosis (Medeiros et al., 2017). Signal transducer and activator of transcription 3 (STAT3), a member of STAT family, is a transcription factor, which can inhibit the degradation of ECM and regulate myocardial fibrosis (Wang et al., 2019; Singh et al., 2021). Therefore, STAT3–MMP9 are promising antifibrosis targets for HF therapeutic strategies.

Phosphatidylinositol three kinase (PI3K)-serine/threonine kinase (AKT) is an important signaling pathway that can protect the heart against cardiac injuries (Wu et al., 2016). PI3K belongs to a conserved family of lipid kinases and is the primary regulator of AKT activation (Chong et al., 2015). It is reported that PI3K could phosphorylate IKK into active form and subsequently activate downstream target NFκB to activate the inflammatory responses (Li et al., 2018). Meanwhile, evidence have shown that activation of PI3K could decrease the level of STAT3 phosphorylation to reduce the fibrosis (Lee et al., 2019). Collectively, the PI3K–AKT signaling pathway plays a crucial role in inflammation response and cardiac fibrosis.

Calycosin (Cal) is one of the major active ingredients in the plant *Astragalus mongholicus* Bunge (Efferth et al., 2016) and has emerged as a highly valued herb to treat cardiovascular diseases. Liu et al. have proven that Cal attenuates myocardial ischemia–reperfusion injury by activating JAK2/STAT3 signaling pathway via the regulation of IL-10 secretion in mice (Liu et al., 2020). Huang et al. have reported that Cal reduces infarct size, oxidative stress, and preserve heart function in an isoproterenol-induced myocardial infarction model (Huang et al., 2020). Zhai et al. revealed that Cal ameliorates doxorubicin-induced cardiotoxicity by suppressing oxidative stress and inflammation via the sirtuin 1-NOD-like receptor protein three pathway (Zhai et al., 2020). However, whether Cal could alleviate MI-induced HF remains unknown. Herein, the left anterior descending (LAD) artery ligation-induced HF rat model was conducted to investigate efficacy evaluation. To explore the mechanism of action of Cal, we applied an RNA-seq approach. Intriguingly, the inflammation and fibrosis pathways are significantly enriched. Furthermore, we performed proinflammatory conditioned media (CM)-induced H9C2 cell lines and TGFβ-stimulated cardiac fibroblasts to verify the precise molecular mechanisms.

## Materials and methods

### Reagents and chemicals

Calycosin was purchased from the Nature Standard Technical Service Co., Ltd. (Shanghai, China). Fosinopril, DMEM, FBS, trypsin, penicillin, streptomycin, sodium cacodylate buffer, and DAPI were purchased from the Beijing BioDee Biotechnology Co., Ltd. (Beijing, China). Paraformaldehyde (4%) and saline (0.9%) were from Applygen Technology Inc. (Beijing, China). Dimethyl sulfoxide (DMSO) was acquired from Sigma-Aldrich LLC (Shanghai, China). LY294002 was purchased from Abmole China Branch. All other chemicals were purchased from commercial sources.

### Animal experiments, grouping, and model establishment

After 1 week of acclimation, Sprague–Dawley (SD) male rats (220 g) obtained from the Beijing Vital River Laboratory Animal Technology Co., Ltd. were randomly divided into four groups (number/each group = 8): sham group, model group, calycosin (Cal) treatment group, and fosinopril treatment group. Rats in the sham group underwent sham surgery, while HF was induced in other rats by direct left anterior descending (LAD) artery ligation as described in our previous study (Zhang et al., 2018). Based on our previous literature (Zhang et al., 2019; Wang et al., 2020a), 24 h after surgery, the acute myocardial infarction model was established, and the drug treatment was started. Rats in the Cal group were treated with Cal at a dosage of 80 mg/kg per day (Li et al., 2020). Rats in the fosinopril group were treated with fosinopril at a dosage of 4.67 mg/kg per day (Wang et al., 2020b). Rats in the sham group and model group were treated with the same volume of distilled water. All the drugs and distilled water were orally administrated with an amount of l ml/100 g for 28 days. It is reported that based on available clinical evidence, fosinopril is an effective and well-tolerated option for the management of patients with heart failure (Davis et al., 1997). For this reason, we set the fosinopril group as a positive control. During the whole procedure, the total mortality of the rats was 30%; most deaths occurred during surgery or after surgery, possibly owing to acute pump failure or fatal arrhythmia. This study conforms to the Guide for the Care and Use of Laboratory Animals published by the US National Institutes of Health (NIH publication no. 85–23, revised 1996), and it was approved by the Institutional Animal Care and Use Committee at the Beijing University of Chinese Medicine (consent number: BUCM20200914-YW).

### Echocardiographic assessment

Transthoracic echocardiography was performed by using a Vevo 2100 instrument (VisualSonics, Canada) equipped with an MS-400 imaging transducer. The echocardiographic measurements were performed under general anesthesia with 1% pentobarbital sodium. M-mode tracings were recorded through the anterior and posterior left ventricular (LV) walls at the papillary muscle level. Heart functions were assessed by related parameters including left ventricular internal dimension—systole (LVID; s), left ventricular internal dimension—diastole (LVID; d), ejection fraction (EF), and fractional shortening (FS). Three cardiac cycles were recorded for the measurements.

### Histological examination

Hearts were cut horizontally through the mid region to create cross sections of both the left and right ventricles, and the apex part was fixed with 4% paraformaldehyde. Then the tissues were embedded in paraffin and cut into 5-μm sections. After deparaffinized by xylene and rehydrated via different grades of ethanol, the sections were stained with hematoxylin–eosin (HE) staining and Masson staining to assess overall pathological changes. Digital images were observed under a microscope at ×400 magnification (Leica Biosystems Richmond, Inc.). Inflammatory cell rate is evaluated by the area of inflammatory cell infiltration using HE staining images. In brief, we selected the region of inflammatory cell infiltration in Image Pro Plus software, then calculated the area of this region as the area of inflammatory cell infiltration of this picture. Then we calculated the area of inflammatory cell infiltration of eight HE views (magnification = original × 400) in the same group. The intensity of inflammatory cell infiltration in other groups was calculated and compared. The collagen volume fraction was also analyzed by Image Pro Plus software in the infarcted border zone. Eight separate images (magnification = original × 400) of Masson staining sections were selected, and collagen volume fraction (CVF) was calculated using the following formula: CVF = collagen area / total visual area × 100%, to assess the degree of cardiac fibrosis.

### Detection of serum biomarkers

The serum was collected from fresh blood and centrifuged at 3,000 × *g* for 10 min at 4°C. The concentrations of serum NTpro-BNP, malondialdehyde (MDA), interleukin-1 (IL-1), and TNFα were detected by enzyme-linked immunosorbent assay.

### Immunohistochemistry assay

Heart sections were deparaffinized and blocked with 5% goat serum for 1.5 h at room temperature. After washing three times with PBS, the sections were incubated with primary antibodies: rabbit polyclonal anti-TNFα antibody (1:500; Abcam; ab220210), rabbit polyclonal anti-collagen I antibody (1:500; Abcam; ab34710), and rabbit polyclonal anti-collagen III antibody (1:500; Abcam; ab7778) at 4°C overnight, and then were incubated with a secondary antibody. Finally, the sections were stained with diaminobenzidine (DAB).

### Ribonucleic acid preparation

In our previous report, RNA-seq was applied in HF rats, the same model as this research (Gao et al., 2020). According to the evaluation results of cardiac function, the difference of intergroup in each group was not significant by comparing with pair-matching *t* test, so we chose three animals per group by random sampling. Total RNA of the cardiac tissues was extracted using TRIzol Reagent^®^ (Invitrogen, Carlsbad, CA, USA). Extracted RNA was digested with dnase to remove contaminating genomic DNA, and the quality of RNA was evaluated by RNA Nano 6000 Assay Kit of the Agilent Bioanalyzer 2100 system (Agilent Technologies, CA, USA). RNA was purified using poly-T oligo-attached magnetic beads, then reverse transcribed into cDNA. After cDNA was ligated with adaptors, PCR amplification was applied with Phusion High-Fidelity DNA polymerase, universal PCR primers, and index (X) primer, and build the library of each sample. The library was sequenced on Illumina Hiseq4000 platform and 150-bp paired-end reads. Quality control and alignment were performed with rat reference sequences. Subsequently, read counts of each gene were computed as raw gene expression.

### Differential expression analysis; GO, reactome, and KEGG pathway enrichment

Using the R package “DESeq2,” differentially expressed genes (DEG) were identified with a *p*-value <0.05 and |log2 (foldchange)| >1 in the R version 3.5.1 software. Furthermore, GO, reactome, and KEGG pathway enrichment were performed using the R package “clusterProfiler” with *p* < 0.05 (R version 3.5.1 software).

### H9C2 cell culture and cell viability

Cell used in the present study were purchased from the China Infrastructure of Cell Line Resources (Institute of Basic Medical Sciences, Chinese Academy of Medical Sciences) and cultured in Dulbecco’s modified Eagle medium (DMEM, Hyclone, USA) supplemented with 10% fetal bovine serum (FBS, Corning, USA), as well as a mixture of penicillin (100 U/ml, Corning, USA) and streptomycin (100 μg/ml, Gibco, USA) at 37°C in a humidified atmosphere of 5% CO_2_. To evaluate the cytotoxicity of Cal in H9C2 cells, cells were cultured in 96-well plates at a density of 6 × 10^3^ cells/well and subjected to different concentrations (2.5, 5, 10, and 20 μM) of Cal treatments. Referring to the inflammatory cell model in our previous study (Li et al., 2016), RAW264.7 cells were subjected to lipopolysaccharide (LPS) (1 μg/ml) for 24 h. Then cell supernatants for conditioned media (CM) were collected for the next experiments. To investigate the effects of Cal on CM-stimulated cardiomyocytes, H9C2 cells were precultured with Cal for 6 h, then stimulated with CM (with/without Cal) for 24 h. CCK-8 was applied to detect the cell viability at 450 nm under a microplate reader.

### Isolation and cardiac fibroblast culture

Cardiac fibroblasts were isolated from neonatal SD rats using mixed enzymatic digestion (0.06% trypsin/0.04% collagenase type Ⅱ in D-PBS without Ca^2+^/Mg^2+^). Then fibroblasts were separated from cardiomyocytes by differential adhesion for about 90 min. The adherent cells are fibroblasts. Cells at passages 2–6 were cultured with serum-free media for about 24 h and subsequently stimulated with 20 ng/ml of TGF-β1 (PeproTech, USA) and Cal or LY294002 for 24 h.

### Detection for supernatant biomarkers

Levels of tumor necrosis factor-α (TNF-α) and IL-1 in cell supernatant were assessed by following the protocols of commercially available kits (NanjingJiancheng, China). The content was expressed as pg/ml.

### Evaluation of mitochondrial transmembrane potential

Mitochondrial membrane potential (MMP) in different groups were evaluated by the JC-1 staining kit (Beyotime Biotechnology, China). H9C2 cells were cultured on laser confocal dishes and then induced by CM. Different groups of H9C2 cells were incubated with JC-1 probe for 30 min at 37°C in the dark. After washing three times with PBS, the images were scanned by laser confocal microscopy (Leica Microsystems GmbH). Image ProPlus (IPP) software was applied to calculate and analyze the ratio of aggregates/monomers fluorescence intensity.

### Detection of reactive oxygen species

ROS was assessed by a commercial assay using fluorescent probe DCFH-DA. H9C2 cells were induced by CM in the presence or absence of Cal and LY294002, and then observed at excitation and emission wavelengths of 488 and 525 nm under a fluorescence microscope (Leica Microsystems GmbH).

### Cell immunofluorescence

H9C2 cells were grown onto confocal dishes for the specified experiment time, fixed with 4% paraformaldehyde for 12 min followed by 0.5% Triton X-100 for 20 min, and blocked with normal goat serum for 1.5 h. Then cells were incubated with NFκB antibody overnight at 4°C. After three times of washing, cells were incubated with the secondary antibody at room temperature for 1.5 h in the dark. After being washed three times, cells were counterstained with 5 μg/ml of DAPI for 20 min. Images were then obtained under a confocal microscope. For α-SMA immunofluorescence, anti-α-SMA antibody (1A4, Santa Cruz Biotech) and an Alexa–Fluor 488-labeled secondary antibody (Molecular Probes) were used.

### Western blot analysis

Heart tissues and H9C2 cells were homogenized in RIPA lysis buffer and quantified by the bicinchoninic acid (BCA) method. A total of 50 μg of protein was separated by 10% sodium dodecyl sulfate polyacrylamide gel electrophoresis (SDS-PAGE) gel and then transferred to a PVDF membrane. After being blocked in a solution of TBST with 5% skimmed milk for 1.5 h at room temperature, the membranes were incubated overnight at 4°C with the following primary antibodies: anti-*p*-NFκB (ab97726; Abcam, USA), NFκB (CST8242, Cell Signaling Technology, Germany), antiTNFα antibody (ab183218; Abcam, USA), anti-p-STAT3 (ab76315; Abcam, USA), STAT3 (ab68153; Abcam, USA), MMP-9 (ab38898; Abcam, USA), PI3K (ab182651; Abcam, USA), AKT (ab182729; Abcam, USA), *p*-AKT (CST4060, Cell Signaling Technology, Germany), *p*-IKKα/β (CST2697T, Cell Signaling Technology, Germany), IKKα (CST2682, Cell Signaling Technology, Germany), IKKβ (CST8943, Cell Signaling Technology, Germany), and anti-GAPDH (ab8245, Abcam, 1:5,000) at 4°C overnight. Afterward, membranes were washed and incubated with specific horseradish peroxidase (HRP)-conjugated secondary antibodies (goat antirabbit IgG 1:12,000 and goat antimouse IgG 1:5,000) for 1 h. The blots were visualized with enhanced chemiluminescent (ECL) plus Western blotting detection reagent (GE Healthcare, UK) for 1 min at room temperature without light, and then captured and analyzed by UVP BioImaging Systems (Bio-Rad, Hercules, CA, USA). Furthermore, protein expressions were normalized based on GAPDH level, and grayscale analysis was performed by the Image-Lab software.

### Statistical analysis

Statistical analysis was performed with the SPSS software (SPSS version 22.0) or GraphPad Prism 7. All data were presented as the mean ± standard deviation (SD). Data were carried out by Dunnett’s test and one-way analysis of variance (ANOVA) to compare differences among multiple groups. *p*-Values less than 0.05 were considered statistically significant.

## Results

### Efficacy evaluation after calycosin treatment in heart failure model

**FIGURE 1:**
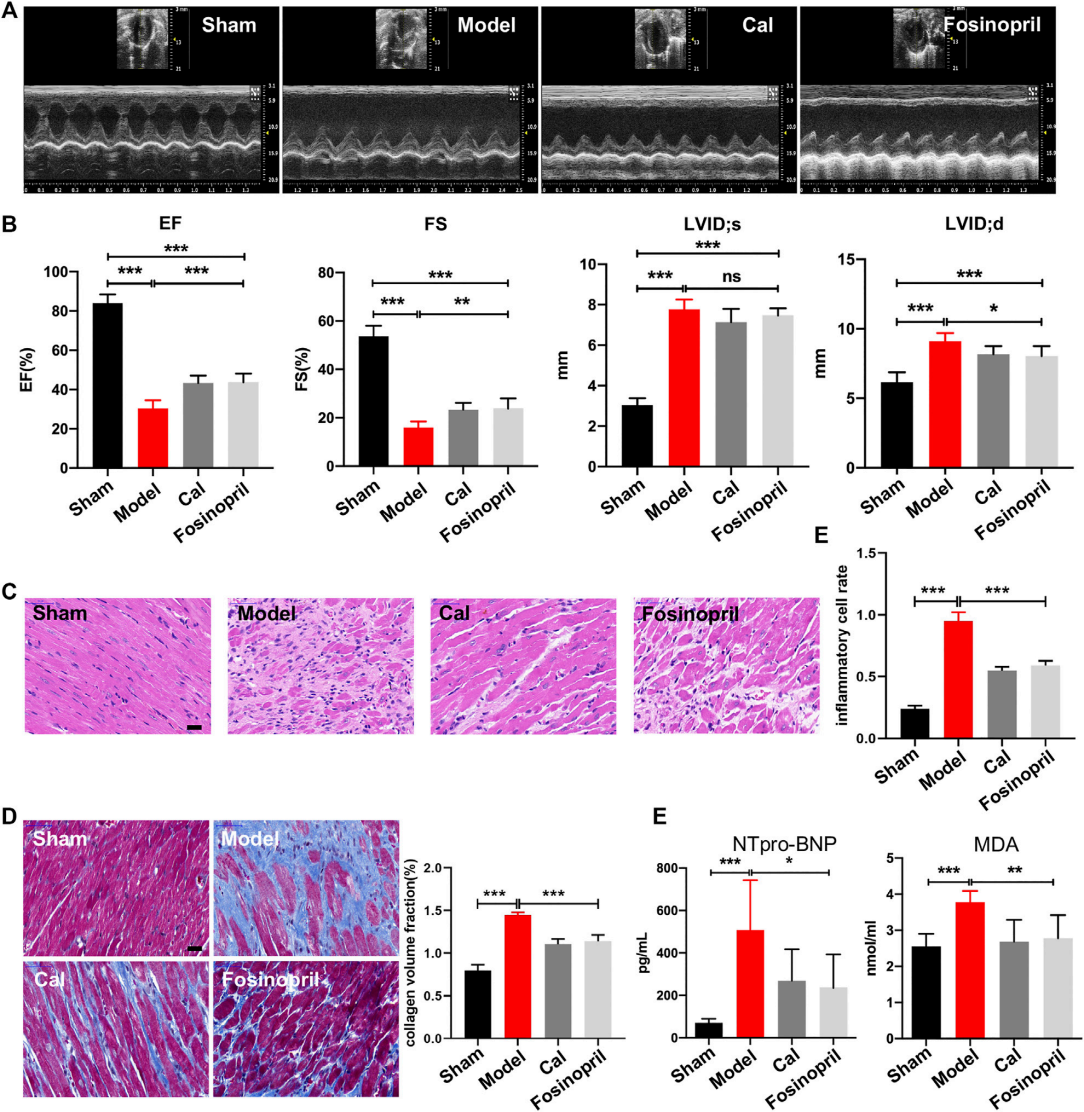
Calycosin (Cal) protected heart function against heart failure (HF) in rats. **(A)** Representative images of echocardiography representing the changes in cardiac function. **(B)** Echocardiographic analysis showed that Cal and fosinopril treatment could increase ejection fraction (EF) and fractional shortening (FS), and decrease left ventricle internal dimension—diastole (LVID; d) values, while there were no significances on left ventricle internal dimension—systole (LVID; s). **(C)** Hematoxylin–eosin (HE) staining showed that Cal and fosinopril treatment protected against the inflammation injury; scale bar = 50 μm. Quantification of inflammatory cell infiltration (%) showed that Cal and fosinopril treatment decreased the inflammatory cell rate. **(D)** Masson staining and quantification of collagen volume fraction (CVF) showed that Cal and fosinopril treatment reduced collagen deposition and alleviated myocardial fibrosis; scale bar = 50 μm. **(E)** Levels of serum NTpro-BNP and MDA in the different groups. Data are presented as the mean ± standard error of three independent experiments (*N* = 8 per group, *p < 0.05, ** *p* < 0.01, and *** *p* < 0.001).

After 28 days of treatments, echocardiography was implemented to examine cardiac function. The results indicated that rats in the model group had significantly lower values of EF and FS, while the diameters on LVID; s and LVID; d were longer than that in the sham group, revealing that the HF model was successfully induced. After treatment with Cal and fosinopril, both EF and FS were increased significantly compared with the model group, suggesting that the left ventricular function was improved by Cal and fosinopril treatment. Additionally, compared with the model group, LVID; d in Cal and fosinopril groups were reduced significantly, while LVID; s in Cal and fosinopril groups were of no statistical significance (Figures 1A, B). The pathological changes in heart tissue were detected by H&E staining. As shown in Figure 1C, the left ventricular areas of the rats in the sham group were clearly visible, with the myocardial fibers arranged neatly and no inflammatory cell infiltration exerted in the myocardial interstitial space. However, in the heart tissue of rats in the model group, a significantly increased infiltration of inflammatory cell was present, myocardial fibers in the infarcted area were almost dissolved, and the myocardial stripes disappeared. Treatment with Cal and fosinopril rescued hearts from inflammatory cell infiltration and improved these pathological changes obviously (Figure 1C). Besides, Masson staining showed that there was obvious extensive collagen deposition in the border zone of the infarction in HF rats, whereas treatment with Cal and fosinopril significantly reduced contents of collagen deposition (Figure 1D). In addition, compared with the model group, Cal and fosinopril significantly reduced the serum levels of NTpro-BNP and MDA, which are the biomarkers of cardiac injury in HF (Figure 1E). These data indicated the protective effects of Cal and fosinopril on cardiac function.

### Identification of differentially expressed genes associated with calycosin treatment

**FIGURE 2:**
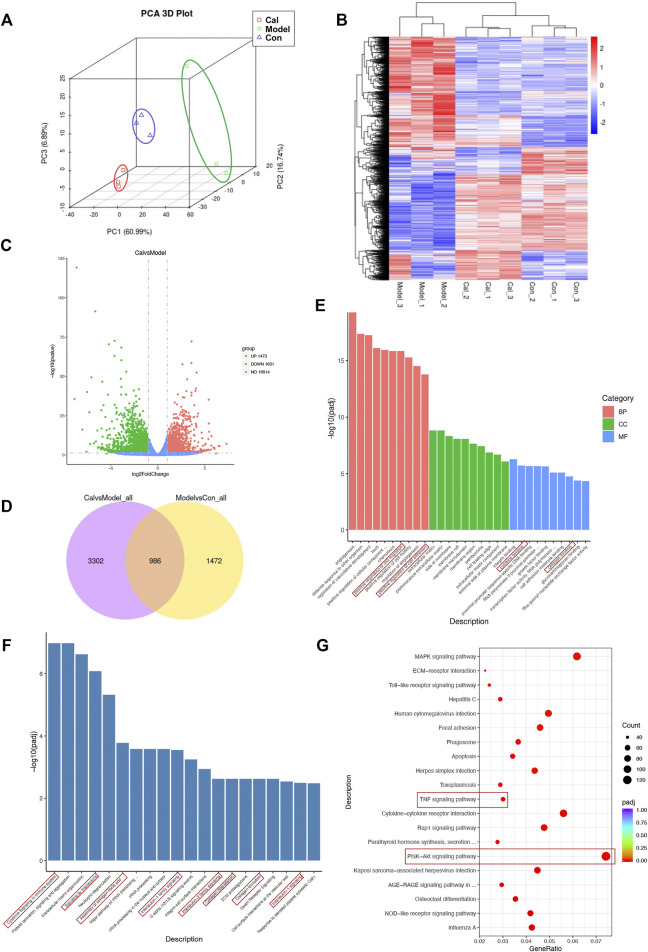
Functional analysis of the differentially expressed genes (DEGs). **(A)** Principal component analysis (PCA) was used for visualizing RNA-seq between the different groups. **(B)** Heatmap of total DEGs between the different groups. **(C)** Volcano plots of DEGs between the Cal-treated group and the model group. The *x*-axis represents log2 (fold change) and the *y*-axis represents −log10 (padj). Red dots represent log2 > 1 and *p-*value < 0.05. Green dots are log2 < −1 and *p*-value < 0.05. **(D)** The 986 common DEGs were identified by Venn diagram. **(E)** The identified DEGs (Cal group vs. model group) were used for various Gene Ontology enrichment analysis including biological process (BP), cellular component (CC), and molecular functions (MF). The identified DEGs (Cal group vs. model group) were used for reactome **(F)** and KEGG **(G)** pathway classification analysis. *N* = 3 per group.

We utilized RNA-Seq to determine the effect of Cal on the cardiac transcriptome. Principal component analysis (PCA) was used for visualizing RNA-seq between different groups (Figure 2A). A hierarchical cluster heatmap between different groups illustrated the differentially expressed genes (DEGs) (Figure 2B). Compared with the model group, a total of 3,104 DEGs (1,473 upregulated and 1,631 downregulated genes) satisfied |log2 (foldchange)| > 1 and the adjusted *p*-value less than 0.05 in the Cal-treated group (Figure 2C). Besides, the 986 key targets of Cal treatment against the HF model were obtained by overlapping the 4,288 targets of Cal treatment and 2,458 targets of HF model with a Venn diagram (Figure 2D). Furthermore, the identified 3,104 DEGs (Cal group vs. model group) were further used for Gene Ontology and functional pathway enrichment analysis. Intriguingly, enrichment analyses of GO annotations and reactome pathways revealed that the DEGs are related to inflammatory response and fibrosis in the Cal treatment group compared with the model group (Figures 2E, F). Besides, KEGG enrichment analysis of 3,104 DEGs showed that the involved pathway of Cal treatment was mainly composed of the PI3K–Akt signaling pathway, TNF signaling pathway, cytokine–cytokine receptor interaction, and MAPK signaling (Figure 2G). In conclusion, the integrated RNA-seq results suggested that the cardioprotective effects of Cal may owe to anti-inflammation and antifibrosis.

### Effects of calycosin on myocardial inflammation via IKKs-NFκB pathway

**FIGURE 3:**
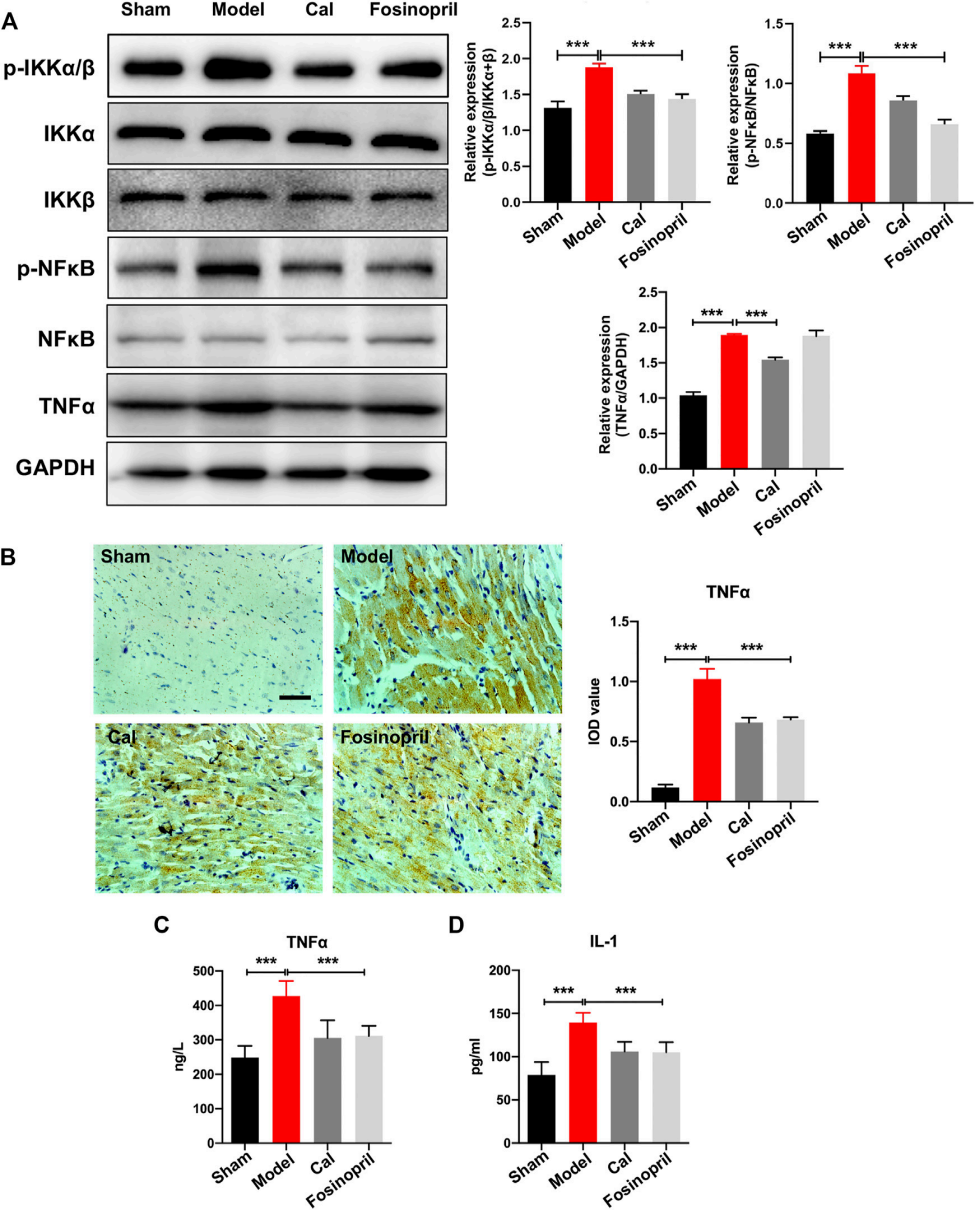
Effects of Cal on myocardial inflammatory via IKKs-NFκB pathway. **(A)** Western blot bands of *p*-IKKα/β, *p*-NFκB, and TNFα and their quantitative results in HF rats, Cal could downregulate the expressions of *p*-IKKα/β, *p*-NFκB, and TNFα, while fosinopri did not decrease *p*-NFκB expression. **(B)** IHC images of TNFα and quantitative results in different groups. Levels of serum TNFα **(C)** and IL-1 **(D)** in the different groups. Data are presented as the mean ± standard error of three independent experiments. (*N* = 3 per group, ** *p* < 0.01, and *** *p* < 0.001).

The IKKs-NFκB pathway is considered as the initiation of inflammatory cascade. WB results implied that expressions of IKKα/β and NFκB remained relatively unaltered among different groups, while the expression levels of phosphorylated IKKα/β (*p*-IKKα/β) and NFκB (*p*-NFκB) increased significantly in the model group. After treatment with Cal, the expressions of *p*-IKKα/β and *p*-NFκB were reduced significantly, while only *p*-NFκB was decreased markedly in the fosinopril group (Figure 3A). The expression of downstream target activated by NFκB was further detected. Protein level of TNFα in the model group was increased, whereas Cal and fosinopril treatment suppressed the expression of TNFα (Figure 3A). Additionally, immunohistochemistry results showed that IOD of TNFα in the model group was upregulated significantly. After treatment with Cal and fosinopril, IOD of TNF was decreased (Figure 3B). Besides, Cal and fosinopril significantly reduced the serum levels of TNFα (Figure 3C) and IL-1 (Figure 3D). Collectively, these data indicated that Cal and fosinopril could alleviate inflammatory responses in HF rats.

### Effects of calycosin on myocardial fibrosis via STAT3-MMP9 pathway

**FIGURE 4:**
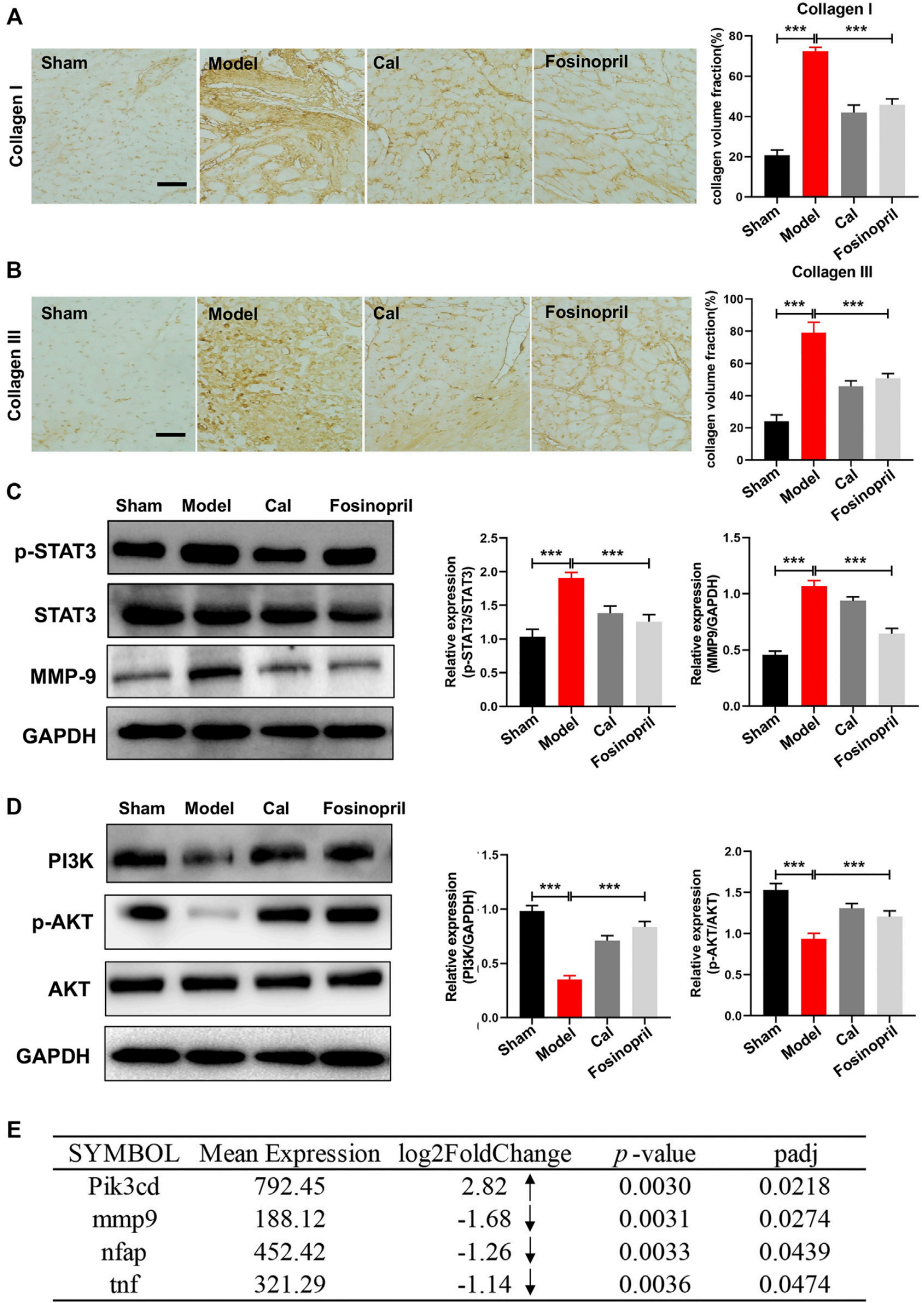
Effects of Cal on myocardial fibrosis via the STAT3–MMP9 pathway. IHC images of collagen Ⅰ **(A)** and Ⅲ **(B)** in HF rats. Quantitative results of types Ⅰ and Ⅲ collagen content with IOD value; Cal and fosinopril could significantly reduce the collagen deposition compared with the model group. **(C)** Western blot bands of p-STAT3 and MMP-9 and their quantitative results in HF rats; Cal and fosinopril could downregulate the expressions of p-STAT3 and MMP-9. **(D)** Western blot bands of PI3K, *p*-AKT, and AKT and their quantitative results in HF rats. **(E)** The differentially expressed genes between the Cal group and the model group. The arrows represent the upregulated or downregulated expression of gene. Data are presented as the mean ± standard error of three independent experiments. (*N* = 3 per group, **p < 0.01, ***p < 0.001).

To investigate the effects of Cal and fosinopril on antifibrosis, the contents of collagen I and Ⅲ in the cardiac tissue were determined by IHC. Results showed that IOD of collagen Ⅰ and Ⅲ in the model group were increased, compared with that in the sham group. After treatment with Cal and fosinopril, IODs of collagens Ⅰ (Figure 4A) and Ⅲ (Figure 4B) were both reduced. These results demonstrated that Cal and fosinopril could effectively attenuate cardiac fibrosis through the inhibition of expressions and depositions of collagen I and collagen III. Recent evidence indicates that the sustained activation of STAT3 signaling after MI may contribute to adverse remodeling and progression to heart failure (Nural-Guvener et al., 2015). MMP-9 is involved in post-MI repair and remodeling by regulating ECM metabolism and processing inflammatory mediators (Frangogiannis, 2017). The results showed that expressions of phosphorylated STAT3 (p-STAT3) and MMP-9 were upregulated in the model group, and Cal and fosinopril impressively inhibited the expressions of p-STAT3 and MMP-9 compared with the model group (Figure 4C).

PI3Ks are kinases that are responses for different types of membrane receptors, which have been observed to be activated in many cardiovascular diseases such as heart failure, hypertension, and atherosclerosis (Ghigo et al., 2013). *In vivo* results implied that the expressions of PI3K and *p*-AKT in the model group were decreased, while after treatment with Cal and fosinopril, the expressions were both upregulated remarkably (Figure 4D). Besides, transcriptome analysis revealed that the mRNA level of Pik3cd was significantly increased under Cal treatment, compared with the model group (Figure 4E). The Cal treatment dramatically downregulated the mRNA levels of mmp9, tnf, and nfap (NFκB activating protein), respectively (Figure 4E).

### Anti-inflammation effects of calycosin via PI3K–AKT signaling pathway in cardiomyocytes

**FIGURE 5:**
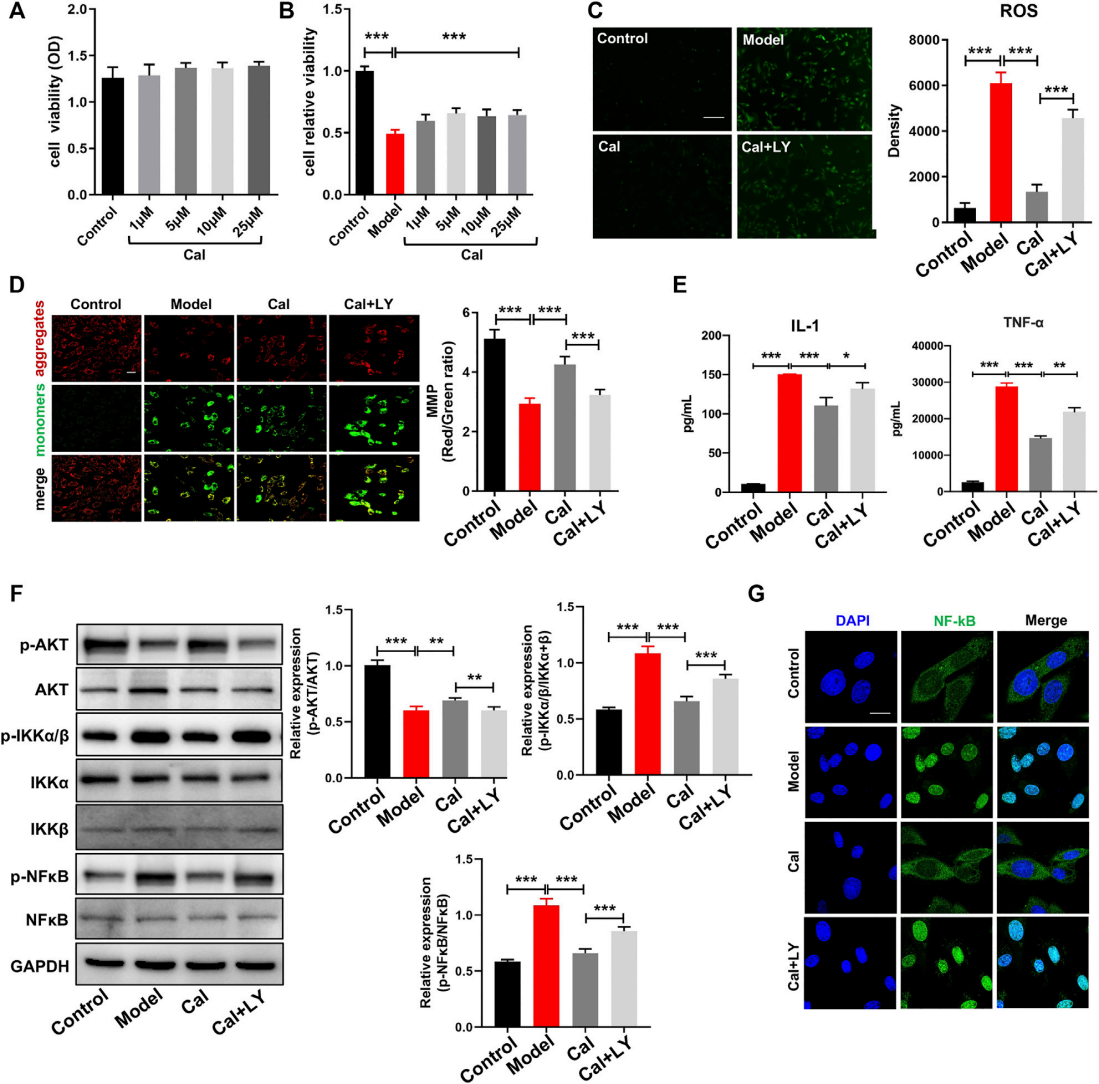
Anti-inflammation effects of Cal via the PI3K–AKT signaling pathway in cardiomyocytes. CCK8 assay was used to determine cell injury. **(A)** Cal (1–25 μM) has no cytotoxicity on H9C2 cells compared with the control group. **(B)** Conditioned media (CM)-induced cellular injury in H9C2 cells and Cal (5 μM) increased cell viability significantly. **(C)** reactive oxygen species (ROS) staining in the different groups, *N* = 12. (D) Mitochondrial membrane potential (MMP) was determined by JC-1 probe (400×, scale bar = 100 μm), *N* = 12. **(E)** The release of IL-1 and TNF-α in H9C2 cell supernatants was detected by ELISA assay, *N* = 12. **(F)** Western blot bands of *p*-AKT, *p*-IKKα/β, and *p*-NFκB and quantitative results in cells. **(G)** Immunofluorescence staining of NFκB under different treatments; scale bar = 50 μm. *N* = 20 cells per group. Data are presented as the mean ± standard error of three independent experiments. *p < 0.05, **p < 0.01, ***p < 0.001.

To further confirm the regulatory mechanism of Cal on inflammation and to better simulate the pathological environment of cardiomyocytes under inflammatory condition in HF, we applied a macrophage conditioned media (CM)-stimulated cardiomyocyte model described in our previous study (Li et al., 2016). Cell viability was reduced dramatically, and cell injury had occurred as characterized by induction of ROS and mitochondria damage. H9C2 cell, an embryonic cardiomyocyte cell line, was selected here owing to its robust and fast reaction to various stimuli. As shown in Figures 5A, B, treatment with 1–25 μM Cal proved to be effective, and 5 μM Cal showed the best protective effect on cell viability. So, 5 μM Cal was the optimal concentration applied in the subsequent experiments. During the inflammatory phase of heart failure, myocardial cell death and hypoxia trigger the overgeneration of reactive oxygen species (ROS) and the damage of the mitochondria. ROS staining showed that Cal dramatically reduced the level of ROS (Figure 5C). The evaluation of mitochondrial transmembrane potential (MMP) was conducted by JC-1 probe (Zhang et al., 2021). The results suggested that the ratio of aggregates/monomers increased in response to Cal, suggesting that the MMP returned to normal (Figure 5D). Besides, Cal significantly reduced the release of TNFα and IL-1 (Figure 5E). Intriguingly, LY294002, an inhibitor of PI3K, compromised these protective effects of Cal in CM-induced H9C2 cells.

As the PI3K–AKT pathway plays a vital role in regulating the inflammation, we compared the expression levels of *p*-AKT, *p*-IKKα/β, and *p*-NFκB in CM-induced H9C2 cells with or without Cal treatment. WB results implied that expression of *p*-AKT was impressively reduced in the model group, while treatment with Cal could promote the expression of *p*-AKT (Figure 5F). Besides, the expressions of *p*-IKKα/β and *p*-NFκB were both markedly increased in the model group compared with the control group, while treatment with Cal downregulated their expressions, respectively (Figure 5F). Immunofluorescence results also showed that Cal treatment inhibited nuclear translocation of NFκB (Figure 5G). To further explore the anti-inflammation effect of Cal on the PI3K–AKT pathway, LY294002, an inhibitor of PI3K, was added together with Cal. Intriguingly, LY294002 suppressed the expression of *p*-AKT. Furthermore, the regulation on *p*-IKKα/β, NFκB activation, ROS level, MMP, and proinflammatory cytokine level by Cal was also eliminated by LY294002 (Figures 5D–G), indicating that Cal protected against CM-induced injury in H9C2 cells partly by targeting on the PI3K–AKT pathway. Collectively, these results demonstrated that Cal could alleviate inflammation by activating PI3K–AKT pathway in cardiomyocytes.

### Antifibrosis effects of calycosin via PI3K–AKT signaling pathway in cardiac fibroblasts

**FIGURE 6:**
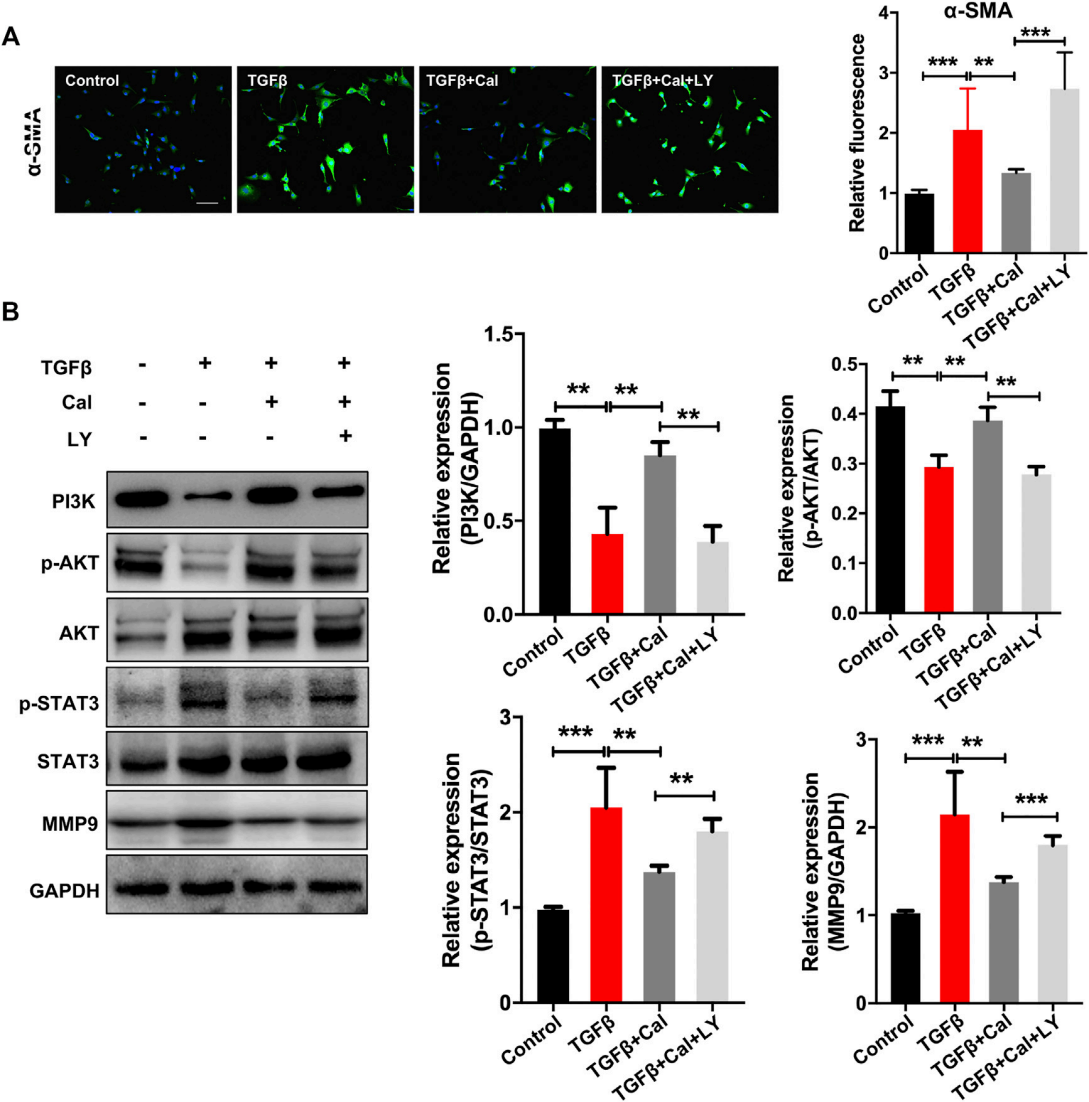
Cal regulated the TGFβ-activated PI3K–Akt signaling pathway in cardiac fibroblasts. **(A)** Representative images of α-SMA immunoassay in each group. Scale bar = 50 μm. *N* = 20 cells per group. **(B)** Western blot bands of PI3K, AKT, STAT3, and MMP9 and their quantitative results in cells. Data are presented as the mean ± standard error of three independent experiments. *p < 0.05, **p < 0.01, ***p < 0.001.

The effects of Cal on TGFβ-stimulated cardiac fibroblasts were further investigated. Cardiac fibroblasts were incubated with TGFβ at a concentration of 20 ng/ml for 24 h. TGFβ induces transformation of fibroblast to myofibroblast, which is the main producer of collagens and is characterized by the presence of α-smooth muscle actin (α-SMA). Our results showed that α-SMA expression was increased in TGFβ-stimulated cells, suggesting that fibroblasts were phenotypically transformed into myofibroblasts (Figure 6A). Cal treatment suppressed TGFβ-induced expression of α-SMA, dramatically reduced the expressions of p-STAT3 and MMP-9, and upregulated the expressions of PI3K and *p*-AKT compared with the model group (Figures 6A, B). While cotreatment with LY294002 abolished these effects. These data suggested that Cal could block the transformation of fibroblast to myofibroblast, thereby suppressing cardiac fibrosis via the PI3K–AKT pathway.

## Discussion

The main goal of this research was to explore the relationship between the PI3K–AKT signaling pathway and the potential anti-inflammation/antifibrosis effects of Cal on HF in rats and in H9C2 cells. Our study provided a novel approach and mechanism for the treatment of HF. The main findings are as follows: 1) Cal ameliorated cardiac functions and alleviated pathological changes in an HF rat model. 2) Cal inhibited excessive release of proinflammatory cytokines by inhibiting IKK-NFκB-mediated inflammatory pathway in cardiomyocytes. 3) Cal blocked the transformation of fibroblast to myofibroblast through the inhibition of STAT3-MMP9 signaling. 4) These regulative effects were accessed by targeting the PI3K–AKT pathway.

HF is typically associated with myocardial fibrosis. Here, inflammation and fibrosis are thought to play critical roles. Activation of an inflammatory reaction and the subsequent secretion of inflammatory cytokines, such as TNFα, facilitate the recruitment and activation of more inflammatory cells to the developing lesion (Huang and Frangogiannis, 2018). Cardiac fibrosis is characterized by an increased amount and a disrupted composition of the fibrillar/collagen-rich ECM. Excessive fibroblast activation may lead to expansion of the fibrotic scar area, which increases myocardial stiffness and promotes diastolic dysfunction (Kong et al., 2018). In our HF rat model, levels of TNFα and IL-1 elevated significantly. In addition, cardiac remodeling characteristics were observed, such as cardiomyocyte derangement, infiltrated inflammatory cells, as well as abnormal deposition of collagen. Therefore, inflammation and fibrosis are interesting targets for innovative heart failure treatments. The exciting part is that Cal treatment rescued hearts from inflammatory cell infiltration and maintained original morphology as well as reduced contents of collagen deposition in HF models. Furthermore, Cal treatment could reverse the abnormal elevation of serums TNFα level and IL-1 level in HF rats.

To investigate the potential mechanisms of Cal, RNA-seq is applied. GO significant enrichment analysis identified that anti-inflammation and antifibrosis effects are involved in Cal treatment, and the PI3K–AKT signaling pathway is regulated by Cal to ameliorate the cardiac function of HF according to KEGG enrichment analysis. A few studies have reported changes in PI3K signaling in HF (Ghigo et al., 2017; Durrant and Hers, 2020), There is controversy in the literature as some studies reported an activation, while others reported an inhibition of PI3K (Hao et al., 2020). Collectively, our findings can give a deeper insight on the therapeutic mechanisms of Cal in treating HF.

To further investigate the underlying mechanism of anti-inflammation and antifibrosis, a CM-induced H9C2 cell model and a TGFβ-induced cardiac fibroblast model were conducted, respectively. In cardiomyocytes, Cal significantly improved cell viability, inhibited ROS production, and restored MMP against CM-induced injury. Besides, results showed that Cal could downregulate levels of *p*-IKKα/β, *p*-NFκB, TNFα, and IL-1, demonstrating that Cal has a regulative efficacy on anti-inflammation pathway in cardiomyocytes. In cardiac fibroblasts, Cal showed a marked effect on myocardial fibrosis evidenced by decreased α-SMA expression. Besides, Cal could decrease the protein levels of p-STAT3 and MMP-9. These results indicated that Cal could alleviate inflammatory responses and attenuate cardiac fibrosis to exert cardioprotection against HF. The upstream pathways of inflammation and fibrosis were further demonstrated. Cal could enhance the expression of PI3K, then activate *p*-AKT expression dramatically. LY294002, an inhibitor of PI3K, was then added together with Cal, to further validate the effect of Cal on the PI3K–AKT pathway. Results indicated that the protective effects and relative protein levels of *p*-AKT, *p*-IKKα/β, and *p*-NFκB were abrogated by LY294002 in cardiomyocytes. In addition, cotreatment with LY294002 also abolished the effects of Cal on α-SMA, p-STAT3, and MMP9 levels in cardiac fibroblasts. Collectively, these data indicated that Cal exerted anti-inflammation and antifibrosis effects against HF through the PI3K–AKT pathway.

## Conclusion

**FIGURE 7:**
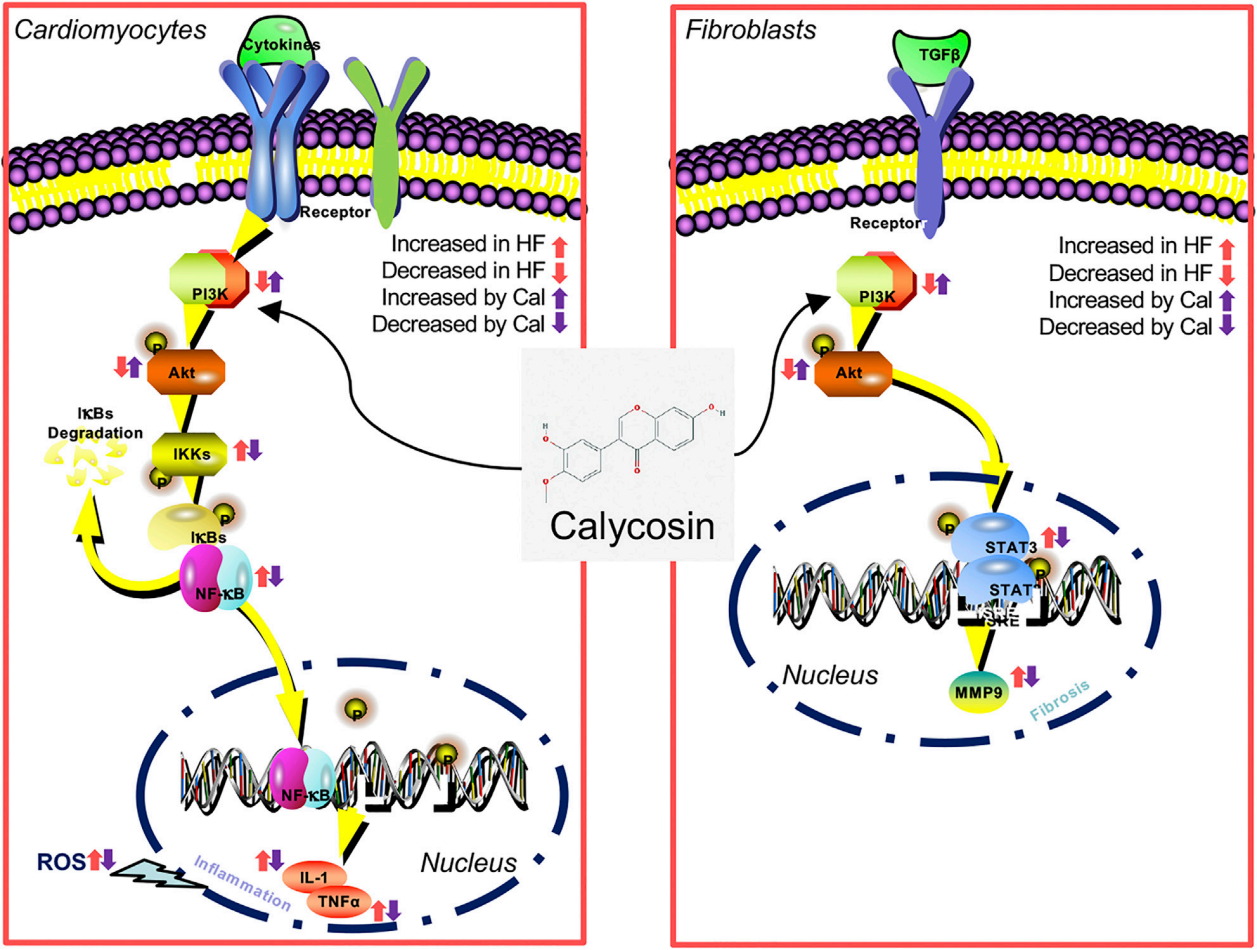
A diagram of the involvement of PI3K–AKT in the inhibition by Cal of cardiac inflammation and fibrosis induced by myocardial infarction (MI).

This study implies that Cal reduces cardiac inflammation and fibrosis via the PI3K–AKT signaling pathway in H9C2 cells, fibroblasts, and heart failure postacute myocardial infarction rats (Figure 7). Cal, as a novel PI3K activator, reduces inflammation and fibrosis in heart failure through the AKT–IKK/STAT3 axis. These findings aid our understanding on the therapeutic mechanisms by which Cal exerts cardioprotection.

Our work still includes some limitations. In our previous study, 1–10 μg/ml of LPS had no effects on the production of NO and LDH in H9C2 cells, indicating that LPS is not an ideal agent for inducing inflammation in H9C2 cells (Li et al., 2016). As cytokines released from macrophages play an important role in the progression of inflammation in ischemic heart tissue (Li et al., 2016), we concluded that cytokines contained in the conditioned medium used to stimulate H9C2 cells is critical. To determine whether the conditioned medium contain LPS is important, and we would perform mass spectrometry analysis in our future study. Since TGFβ is not an equivalent stimulus to the *in vivo* HF model. In order to replicate the *in vivo* and HF phenotype more appropriately, methodologically, we will use fibroblasts cultured from sham rats and HF rats to make our conclusions much more compelling and powerful. Besides, we will include the fosinopril group in the RNA seq and *in vitro* data. In addition, we will carry out experiments to explore whether LY can blunt the impact of Cal *in vivo*.

## Data Availability

The datasets presented in this study can be found in online repositories. The names of the repository/repositories and accession number(s) can be found below: https://www.ncbi.nlm.nih.gov/, https://www.ncbi.nlm.nih.gov/geo/query/acc.cgi?acc=GSE184649.
